# Minimally Invasive Intracerebral Hemorrhage Evacuation Techniques: A Review

**DOI:** 10.3390/diagnostics11030576

**Published:** 2021-03-23

**Authors:** Theodore C. Hannah, Rebecca Kellner, Christopher P. Kellner

**Affiliations:** Department of Neurosurgery, Icahn School of Medicine at Mt Sinai, New York, NY 10029, USA; rebecca.kellner@icahn.mssm.edu (R.K.); christopher.kellner@mountsinai.org (C.P.K.)

**Keywords:** minimally invasive surgery, intracerebral hemorrhage, SCUBA, MISTIE, ICH evacuation, neurosurgery

## Abstract

Intracerebral hemorrhage (ICH) continues to have high morbidity and mortality. Improving ICH outcomes likely requires rapid removal of blood from the parenchyma and restraining edema formation while also limiting further neuronal damage due to the surgical intervention. Minimally invasive surgery (MIS) approaches promise to provide these benefits and have become alluring options for management of ICH. This review describes six MIS techniques for ICH evacuation including craniopuncture, stereotactic aspiration with thrombolysis, endoport-mediated evacuation, endoscope-assisted evacuation, adjunctive aspiration devices, and the surgiscope. The efficacy of each modality is discussed based on current literature. The largest clinical trials have yet to demonstrate definitive effects of MIS intervention on mortality and functional outcomes for ICH. Thus, there is a significant need for further innovation for ICH treatment. Multiple ongoing trials promise to better clarify the potential of the newer, non-thrombolytic MIS techniques.

## 1. Introduction

Intracerebral hemorrhage (ICH) accounts for up to 20% of all strokes with 40,000 to 67,000 cases per year in the US [1,2]. Despite rapid and accurate diagnostic imaging techniques, the associated morbidity and mortality following ICH remains quite high. It has been estimated that only 20% of patients will ever regain full function and 40% die within one month [1,2]. Classically, ICH was been treated with medical management or craniotomy. Craniotomy intervention had been limited to those with superficial lobar or cerebellar bleeds causing significant neurological impairment [3]. The International Surgical Trial in Intracerebral Hemorrhage (STICH I) and STICH II trials compared surgical intervention to medical management, but could not definitively clarify surgery resulted in improved outcomes [3,4,5]. Research into the pathophysiologic mechanisms of ICH has demonstrated that ICH causes damage in two distinct and temporally separate mechanisms. First, the collection of blood within the brain causes mass effects leading to mechanical distortion and increased intracranial pressure (ICP). Elevated ICP leads to mitochondrial injury and aberrant neurotransmitter release. Second, the release of thrombin leads to infiltration of mesenchymal cells, microglia, and inflammatory cells resulting in significant perihematomal edema (PHE) [1]. PHE can cause additional neurological insult and some research asserts that PHE expansion accurately predicts ICH morbidity and mortality, but the true implications of PHE on outcomes remains controversial [6]. Nevertheless, improving ICH outcomes likely requires rapid removal of blood from the parenchyma and restraining edema formation while also limiting further neuronal damage due to the surgical intervention. Minimally invasive surgery (MIS) approaches promise to provide these benefits and, for this reason, have become alluring options for management of ICH. However, the results of rigorous clinical trials of MIS ICH methods over the past decade have been mixed. In addition to describing current methods of ICH diagnosis and prognosis, this article reviews the minimally invasive ICH evacuation methods and the literature describing each of their efficacies.

## 2. Intracerebral Hemorrhage (ICH) Diagnosis and Imaging

Similar to acute ischemic stroke patients, patients with ICH often present with rapid onset of impaired neurological function [1]. Accompanying symptoms can include headaches, seizures, elevated blood pressure, vomiting, and altered level of consciousness. A non-contrast head computed tomography (CT) is the standard neuroimaging technique that provides fast and accurate evaluation for the presence of ICH [2]. Once the diagnosis of ICH is made, clinicians may search for an underlying cause. CT angiography (CTA) is often ordered along with the non-contrast CT to evaluate for vascular pathologies [1].

**Figure 1:**
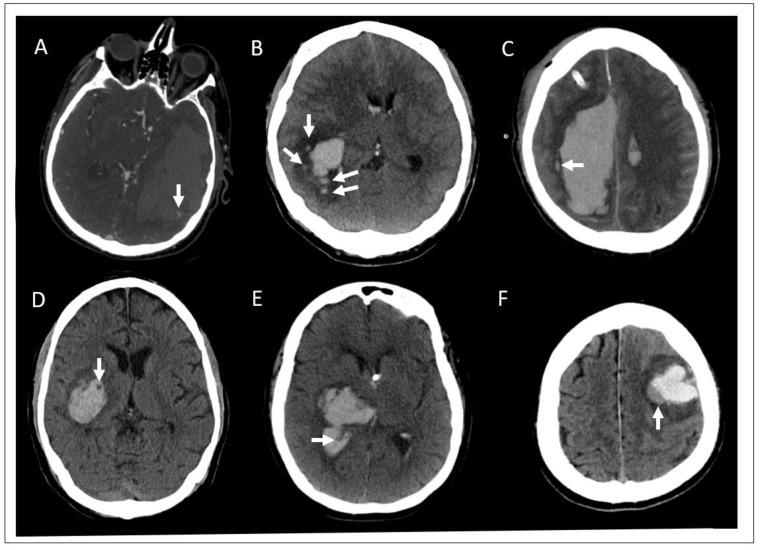
Diagnostic imaging markers associated with hematoma expansion in intracerebral hemorrhage. The various signs associated with hematoma expansion indicated by white arrows in each panel. Spot sign is seen on computed tomography angiography (CTA), the others are found on non-contrast computed tomography head (CTH). (**A**) Spot sign; (**B**) island sign; (**C**) satellite sign; (**D**) black hole sign; (**E**) swirl sign; (**F**) blend sign.

In addition to the presence of hemorrhage and vascular pathologies, clinicians have begun to investigate various patterns and markers on these diagnostic images that predict ICH outcomes, the risk of rebleeding, and the overall benefit of acute surgical intervention. The most well-known imaging biomarker is the spot sign seen on CTA. The spot sign is defined as the presence of a tiny, enhancing foci within the hematoma on CTA caused by contrast extravasation [7]. It is suggestive of active bleeding and thus predicts hematoma expansion. Other signs that have been investigated include satellite sign, island sign, swirl sign, black hole sign, and blend sign (Figure 1). Unlike the spot sign, all of these markers are seen on non-contrast CT scans.

The satellite sign and island sign are both considered shape markers [8,9]. The satellite sign is a small hemorrhage less than 10 mm in diameter and located within 20 mm of the main hematoma. It was present in 37–50% of patients and may be a useful predictor of hematoma expansion and poor functional outcomes, however, a meta-analysis demonstrated only a 50% sensitivity and 71% specificity for hematoma expansion [10,11,12,13]. Island sign is defined as at least three small hemorrhages separated from the main hematoma or at least 4 smaller hemorrhages connected to or separate from the main hematoma. It was present in 15–23% of patients and has also been shown to predict hematoma expansion and poor outcomes [12,14,15,16]. Of note, one comparison study has suggested that the island sign is a better predictor of hematoma expansion than the satellite sign [12]. The swirl sign, black hole sign and blend sign are considered density markers [8,9]. The swirl sign is a low-density area within the hematoma surrounded by a hyper dense region with a poorly defined margin. Studies have found the swirl sign in approximately 30–35% of patients. Although some studies have associated swirl sign with increased hematoma growth, multiple studies have found that it is not an independent predictor of expansion [17,18,19,20]. The black hole sign is a round or oval hypodensity completely surrounded by a hyperdense region with a well-defined border [21]. It was present in 14–22% of patients studied and found to have low sensitivity, but very high specificity, in multiple studies for predicting hematoma expansion and worse functional outcomes [18,21,22,23,24]. The blend sign is a hypodense region within a hematoma that is directly adjacent to, but not encapsulated by, a hyperdense region with a well-defined border [25,26]. It was present in 16–20% of patients and was also shown to be highly specific for early hematoma growth [23,26,27,28,29]. Additionally, one study showed that the combination of the island sign and blend sign accurately predicted hematoma expansion in 92% of cases [25].

Overall, the spot sign has been found to be the most reliable predictor of the risk of hematoma expansion, however, if CTA is unobtainable, the non-contrast CT markers likely provide valuable information about the risk of hematoma growth [23]. Although these advances in ICH imaging interpretation are promising, one caveat researchers must be aware of is that the definition of some signs has varied between studies complicating assessment of those indicators [10,11]. Furthermore, the risks and benefits of surgical versus medical management in the presence of each of these imaging findings requires further study as the trials reviewed in the following sections of this article did not account for these imaging findings when evaluating outcomes or risk of rebleeding of the surgical intervention.

## 3. Minimally Invasive Surgery (MIS) for ICH Techniques

**Figure 2:**
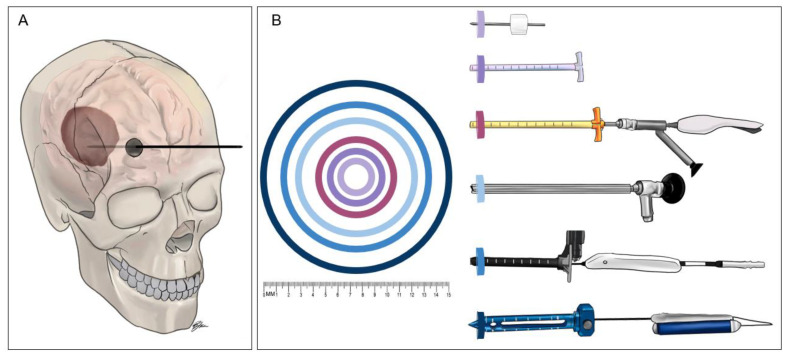
The relative sizes of the instruments of minimally invasive surgery for intracerebral hemorrhage (ICH) evacuation. (**A**) Generic sketch of the minimally invasive approach to intracerebral hemorrhage evacuation. A small craniotomy is made and the chosen device is inserted through the cranial opening and into brain parenchyma until reaching the hematoma. (**B**) Sketches of the ICH evacuation devices with concentric rings demonstrating the widest diameter of the instrument inserted through brain parenchyma for each technique. The color of each concentric ring corresponds to the color at the tip of the device in the illustrations. The devices, from top to bottom, are the craniopuncture YL-1 needle (outer diameter: 3.0 mm), the 14F vascular sheath used in the Minimally Invasive Surgery Plus Rt-PA for ICH Evacuation (MISTIE) procedure (4.8 mm), the Artemis device inserted through a 19F vascular sheath and a 3-port Endoscope such as the Storz Lotta (6.3 mm), the clear sheath used during endoscope-assist procedures (10.0 mm), the Aurora Surgiscope (11.5 mm), and the BrainPath endoport (15.8 mm).

MIS ICH evacuation techniques have many similarities. Surgeons will use imaging reconstruction to determine the best trajectory and corresponding access point to approach the hematoma, taking care to avoid important brain regions and blood vessels. A small access point is made through the skull, then the instrumentation is introduced. Most protocols also require a follow-up computed tomography (CT) scan to determine evacuation efficacy. The differences between techniques are largely in the size of the access port and the instruments used to evacuate the blood clot. Another major point of differentiation is whether the technique involves the infusion of pharmacologic thrombolytics. Finally, there are also differences in the length of time that access to the clot needs to be maintained. Some methods drain the hematoma over days whereas others relinquish access to the clot by the end of the initial surgery. Here, we highlight the use of thrombolytics, the size of the instrumentation and associated burr hole, and when access to the clot is terminated (Figure 2).

## 4. Thrombolytic Techniques

### 4.1. Craniopuncture

Craniopuncture is the standard of care for treating ICH in China [30]. Craniopuncture uses a YL-1 needle which consists of a 3 mm-diameter hollow cannula containing the puncture needle. The puncture needle is drilled through the skull and into the hematoma before the cannula is fixed to the skull and the hematoma is aspirated. Following initial aspiration, a lysis fluid containing urokinase or recombinant tissue plasminogen activator (rtPA) is injected to facilitate further aspiration. The thrombolytic agent is reintroduced into the hematoma every 6–12 h. A follow-up CT scan is performed 1–3 days after initial drainage to measure the amount of blood remaining. The drainage needle remains in the brain for 3–5 days [30,31,32].

The landmark paper for craniopuncture was published in 2009 by Wang et al. [31]. They compared outcomes between craniopuncture and conservative medical management in 377 patients with basal ganglia hemorrhages (25–40 mL in volume) in China (Table 1). The results showed significantly improved neurological function in the craniopuncture group by two weeks with no difference in the rate of rebleeding (9.7% vs. 5.0%). After three months, the percentage of patients with a modified Rankin Score (mRS) > 2 was significantly smaller for those undergoing craniopuncture [31]. However, there was no significant difference in mortality. The following year, Sun and colleagues (2010) published a study demonstrating that craniopuncture also improved outcomes over traditional craniotomy [33]. However, different outcomes were found to be improved. Specifically, there was no improvement in neurological function at three months, but there was a significant decrease in the fatality rate and the rebleed rate (8.8% vs. 21.4%) at 90 days for the craniopuncture. In 2011, Zhou et al. investigated differences between craniopuncture and traditional craniotomy and found no difference in the rebleed rate (10% vs. 15.4%), or fatality rate at one year, but did show improvement across Glasgow Outcome Scale (GOS), mRS, and Barthel Index (BI) [34]. Thus, all three studies demonstrated a benefit of the craniopuncture, although whether the craniopuncture improves neurologic outcomes, mortality outcomes, or both, is less clear. Regardless, these results have led to the craniopuncture becoming the standard of care in China and craniopuncture techniques are now being tested in other hemorrhagic diseases such as chronic subdural and epidural hematomas [35,36]. Recently, the craniopuncture was also tested in an early intervention paradigm, defined as surgical intervention within six hours of symptom onset. Intriguingly, it was found that craniopuncture had worse rebleeding rates (40% vs. 19%) and functional outcomes compared to craniotomy in the presence of the spot sign on CT. However, for patients without the spot sign, craniopuncture was equally efficacious as craniotomy had similar a rebleeding rate (12% vs. 17%) [37]. However, there have been no clinical trials for craniopuncture in the US or Europe and thus craniopuncture is not commonly used in these regions. The most common thrombolytic MIS for ICH intervention used in the US is stereotactic aspiration with thrombolysis.

### 4.2. Stereotactic Aspiration with Thrombolysis

The MISTIE trials refined and evaluated the stereotactic aspiration with thrombolysis procedure. After a patient is deemed eligible, they receive a second CT scan to evaluate the stability of the clot at least six hours following their initial diagnostic CT. If the clot proves stable, a trajectory is chosen and the surgeon drills a 1 cm burr hole at the appropriate site. Using image guidance, a 4.8 mm (14F) diameter sheath is stereotactically inserted into the middle of the hematoma’s short axis and at least three quarters of the way along the long axis of the clot. The clot is then manually aspirated with a syringe until resistance is felt at which point a drainage catheter is inserted and the sheath is removed. The catheter is tunneled subcutaneously away from the burr hole and the incision is closed. The catheter is connected to a three-way stopcock to allow for injection of thrombolytics and saline in addition to drainage. Injections of rtPA start six hours after a post-operative CT scan demonstrates periprocedural clot stability. rtPA is then injected every eight hours and up to nine injections can be given. Injections are stopped after the ninth administration or once the clot is reduced to less than 15 mL. CT scans are performed daily to measure remaining clot and assess clot stability. The catheter is allowed to drain for 24 h after sufficient reduction of the clot or 25 h after the last does of rt-PA before it is removed [30,32,39,42].

Prior to MISTIE, the Stereotactic Treatment of Intracerebral Hematoma by Means of a Plasminogen Activator (SICHPA) trials compared stereotactic aspiration with administration of urokinase to conservative management [38]. The results of this trial, published in 2003, showed no beneficial effect of this surgical technique on mortality or functional outcomes at 180 days. Since then, three phases of the MISTIE trials comparing stereotactic aspiration with thrombolysis to medical management have been published. Initial reports in 2008 suggested that the MISTIE protocol enhanced clot volume reduction over medical management [43]. Another study published at that time further suggested that introduction of the thrombolytic agent did not exacerbate PHE, which had been a significant concern [44]. In 2016, MISTIE-II trial results demonstrated that the MISTIE technique had similar safety outcomes as medical management, that MISTIE may improve mRS outcomes at 180 days likely due to an increase in volume reduction, and that it appeared the procedure reduced PHE relative to the amount of hematoma removed [41]. However, the 72 h rebleed rate was significantly higher using the MISTIE technique (27.8% vs. 9.5%). The efficacy results of MISTIE-III were published in 2019 [39]. There was no significant difference in the primary outcome of mRS at the one-year timepoint. However, there was significant improvement in mortality at one year for the surgical intervention group and, for those patients who achieved hematoma volume reduction to below 15 mL, there was a significant improvement in mRS at one year. Although, the authors caution that these results are exploratory and some have noted that these results may be due to statistical chance [39,45]. A review of less rigorous studies of stereotactic aspiration with thrombolysis has revealed divergent results. For example, one study showed that it is less effective than craniotomy and endoscopic surgery in evacuating hematoma from cerebellar hemorrhages [46]. Other studies have demonstrated that this technique has better clinical outcomes in comparison to craniotomy for basal ganglia hemorrhage [47]. Meanwhile, a meta-analysis of studies comparing stereotactic aspiration with thrombolysis to craniotomy found improved mortality outcomes and lower rebleed rates for stereotactic aspiration (9% vs. 18%) [48]. Nevertheless, the results of the MISTIE trial strongly support the need to evaluate other MIS ICH evacuation techniques.

## 5. Non-Thrombolytic Techniques

### 5.1. Endoport-Mediated Evacuation

The endoport-mediated evacuation method requires a 2.5–3 cm craniotomy at the access point and a subsequent 1.5–2 cm opening in the dura. The BrainPath endoport sheath (Nico Corp, Indianapolis, IN, USA) has a 15.8 mm diameter. Along with the inner obturator, the sheath is inserted through the access point to the deepest part of the clot. The obturator is then removed from the sheath providing surgeons with direct access to the hemorrhage. Removal of the clot can be performed with the Myriad handpiece (Nico Corp, Indianapolis, IN, USA), or common microsurgical tools. Coagulation of vessels and cavity irrigation with normal saline are also performed following evacuation of the clot. Upon completion of this procedure, the endoport is immediately removed [30,32,49,50].

The Early Minimally-Invasive Removal of ICH (ENRICH) trial, investigating the benefits of endoport-mediated evacuation using the BrainPath and Myriad system is currently ongoing and the results are currently unknown. However, there have been a few preliminary studies investigating the efficacy of the BrainPath system. In a retrospective analysis of 11 patients, Przybylowski et al. (2015), found that the endoport permits at least 75% volume reduction of hematoma in most patients which improved mass effect, but outcomes were not compared to a control group [49]. Bauer et al. (2017) described their experience with BrainPath in 18 ICH patients over two years at a single institution. They found that endoport-mediated evacuation was safe and effective, but also did not compare outcomes to a control group [50]. Griessenauer et al. (2018) compared five matched cases of endoport-mediated evacuation to endoscopic evacuation and found they had similar evacuation percentages, but that functional outcomes and mortality were poor in both groups [51]. The only multicenter case series was performed by Labib et al. (2017) [52]. In a review of 39 patients, they showed that endoport evacuation removed over 90% of the hematoma in 72% of patients and 52% of patients with available data had an mRS of two or less at the time of assessment, although the time to surgery varied widely throughout the cohort. There was also no control group for comparison. Consequently, it seems our understanding of the benefit of endoport-mediated evacuation may remain limited until the ENRICH trial findings are published.

### 5.2. Endoscope-Assisted Evacuation

The endoscope-assisted evacuation technique combines an endoscope with a multifunctional aspiration cannula working side by side through an access sheath. The endoscope provides visualization while the cannula allows the surgeon to aspirate the clot, irrigate the cavity, and cauterize blood vessels. These two tools are utilized together, often through a 10 mm diameter sheath. The procedure requires a craniectomy of 15–20 mm in diameter. The sheath is then inserted into the clot and the surgeon applies continuous suction while performing multiple rounds of irrigation and cauterizing blood vessels as necessary. The instruments and sheath are removed at the end of the procedure. At times, a drainage catheter is left in place and tunneled away from the incision [30,32,53].

The first randomized study of an endoscope used in ICH evacuation was in 1989. Auer et al. found that endoscopic evacuation led to significantly lower mortality than medical management as well as a higher percentage of patients with minimal to no residual neurological deficits [40]. Since then, multiple other studies have been performed. Within the past decade, Nagasaka et al. (2011) retrospectively reviewed 23 ICH patients treated with endoscopy versus 20 patients treated with craniotomy. Their results suggested increased evacuation rates and improved Glasgow Coma Scale (GCS) by day seven for the endoscopy group compared. One patient treated with craniotomy and zero patients treated with endoscopy had rebleeding [53]. In a retrospective review of 82 endoscope procedures versus 69 craniotomies, Xu et al. (2018) found that endoscope-assisted surgery resulted in a higher evacuation percentage and improved six-month mRS outcomes [54]. It was also associated with a decreased rebleeding rate (2% vs. 8%). However, a similar study by Wang et al. (2015) did not demonstrate comparably improved outcomes at the six month time point [55]. Likewise, Cai et al. (2017) found no difference in functional or mortality outcomes for endoscopy in comparison to craniotomy or stereotactic aspiration, although endoscopy did have greater evacuation percentages [56]. Conversely, another study comparing these three methods found that endoscopy and stereotactic aspiration had greater functional outcomes than craniotomy. It also suggested that endoscopy was a superior technique for patients with large bleeds of greater than 60 mL [57]. None of these three studies found a difference in rebleeding rate. The Intraoperative Stereotactic Computed Tomography-Guided Endoscopic Surgery (ICES) for Brain Hemorrhage arm of the MISTIE trial evaluated the efficacy of endoscopic evacuation. Over 40% had mRS below 4 at 180 and 365 days compared to just over 20% in the medical management group suggesting that endoscopic evacuation may improve functional outcomes [58]. To the best of our knowledge, there are no ongoing clinical trials specifically testing the endoscope-assisted evacuation technique. However, there have been two recent meta-analyses comparing endoscopic-assisted evacuation to craniotomy. In 2017, Ye and colleagues reviewed eight studies with 1327 patients in both randomized and non-randomized trials. They found that, in randomized control trials, endoscopic-assisted methods improved outcomes and reduced the total risk of complications, but had no effect on mortality in comparison to craniotomy [59]. In 2019, Nam et al. reviewed 3 randomized control trials with 289 subjects and showed that endoscopic-assisted techniques decrease mortality and complication rates in comparison to craniotomy [60]. The three studies in Nam et al. were also analyzed in Ye et al., however the inclusion criteria for Nam et al. was more stringent which may account for the divergent results on the effect of endoscopy on mortality. Similar to the results in studies evaluating craniopuncture, most endoscopy studies suggest there is a beneficial effect, but a rigorously conducted clinical trial will be necessary to determine if there is a true effect and which patients are most likely to benefit.

### 5.3. Adjunctive Aspiration Devices

Adjunctive aspiration devices permit enhanced operator control over aspiration strength reducing inadvertent damage to the brain structures encompassing the hematoma [61]. The Artemis System method of ICH evacuation, and its predecessor, the Apollo, are similar to endoscope-assisted techniques. Using the endoscope working channel, they combine an endoscope with the Artemis System (Penumbra, Alameda, CA, USA) into a single tool for aspiration and irrigation. There are multiple methods for evacuation using these devices, however, the stereotactic ICH Underwater Blood Aspiration (SCUBA) method has arguably become the most prominent. SCUBA leverages the Artemis System to maximize clot removal with variable suction and novel clot morcellation capabilities. Additionally, only a 10 mm craniectomy is necessary as the guidance sheath is 6.3 mm in diameter. Once the guide sheath is inserted to within 2 cm of the distal end of the clot, the introducer is removed and the endoscope/Artemis combination device is inserted. The SCUBA protocol has two phases. In the first, suction is turned to 100% while irrigation flow is at 25%. Gentle exploration of the cavity is performed at that depth and all clot is removed. Then the endoscope is retracted back 1cm and clot is removed at this depth. This process repeats until the endoscope reaches the proximal portion of the clot. Next, the suction is decreased to less than 25% of its maximum to decrease the likelihood of trauma to the cavity walls and irrigation is increased to 100%. The infusion of saline prevents collapse of the cavity and permits the surgeon to find any remaining clot to be suctioned or leaking blood vessels to be cauterized. Once all the clot has been removed, the endoscope is removed [30,32,62,63,64].

The Apollo system proof of concept for ICH evacuation was first published in 2014. Since then interest in the use of adjunctive aspiration devices has grown considerably [62,63,64]. As evidence of this, there are currently three ongoing clinical trials investigating the Apollo or Artemis systems including the Minimally Invasive Endoscopic Surgery with Apollo in Patients with Brain Hemorrhage (INVEST) and Artemis in the Removal of Intracerebral Hemorrhage (MIND) studies in the US as well as the Dutch Intracerebral Hemorrhage Surgery Trial (DIST) trial in the Netherlands. Since the start of these trials, further case series have been published including one by Goyal et al. (2018) demonstrating a possible mortality benefit using the Apollo system over best medical management [65]. Most recently, a report of 100 patients undergoing MIS ICH evacuation with either Artemis or Apollo demonstrated that both are effective at reducing hematoma volumes to under 15 mL and, at 6 month follow-up, 46% of the patients had mRS < 4. The rebleed rate was 5% [66]. Although this is the largest sample of patients treated with this ICH evacuation method, there was no comparison group and so the results of the INVEST, MIND and DIST trials are eagerly awaited.

### 5.4. Surgiscope

The newest instrument being used in MIS ICH evacuation is the Aurora Surgiscope System designed by Rebound Therapeutics. Cleared by the United States Food and Drug Administration (FDA) in January of 2019, it is the first disposable, single-use endoscope and has an outer diameter of 11.5 mm. There have not yet been any published case series, however, interest is likely to grow in the near future as the first clinical trials of this device, Minimally Invasive Intracerebral Hemorrhage Evacuation (MIRROR) and Ultra-Early, Minimally Invasive Intracerebral Hemorrhage Evacuation Versus Standard Treatment (EVACUATE), have just recently begun enrolling patients.

## 6. Conclusions

ICH remains a devastating disease and analysis of mortality outcomes form 2000–2010 showed no decline in case fatality rates [67]. This highlights the need for improved interventions and MIS for ICH evacuation techniques remain the most likely candidate to achieve improved mortality outcomes. However, despite many reports of the benefits of various MIS for ICH evacuation methods, the exact nature of the benefit often differs between studies. Moreover, the largest clinical trials have yet to demonstrate definitive effects of surgical intervention on mortality and functional outcomes. Thus, there is a significant need for further innovation for ICH treatment and the multiple ongoing trials including ENRICH, INVEST, and MIND promise to better clarify the potential of the newer, non-thrombolytic MIS techniques.

## Figures and Tables

**Table 1:** Summary of major clinical trials for minimally invasive surgery for intracerebral hemorrhage evacuation techniques.

Study	Completed or Ongoing	Device	Dates of Enrollment	Locations	Number of Subjects	Results
Wang et al., 2009 [31]	Completed	Craniopuncture	January 2003–June 2004	42 centers in China	195 Craniopuncture vs. 182 Conservative medical management	**Mortality:** 6.7% vs. 8.8% (*p* = 0.44) at 90 days **Functional Status:** significant improvement in 90-day Barthel Index (BI) (χ^2^ = 23.13, *p* = 0.0001) **Rebleeding:** 9.7% vs. 5.0%, *p* = 0.08
Sun et al., 2010 [33]	Completed	Craniopuncture	January 2003–July 2005	22 centers in China	159 Craniopuncture with urokinase infusion vs. 145 Craniotomy	**Mortality:** 14.5% vs. 25.0%, (*p* = 0.02) at 90 days **Functional Status:** no difference in 90-day BI (χ^2^ = 4.166, *p* = 0.38) **Rebleeding:** 8.8% vs. 21.4%, *p* = 0.002
Zhou et al., 2011 [34]	Completed	Craniopuncture	2005–2008	China	90 Craniopuncture vs. 78 Craniotomy	**Mortality:** 18.9% vs. 24.4% (*p* = 0.39) at 365 days **Functional Status:** BI = 79.5 vs. 62 (*p* = 0.01), at 365 days **Rebleeding:** 10.0% vs. 15.4%, *p* = 0.29
Stereotactic treatment of intracerebral hematoma by means of a plasminogen activator (SICHPA) [38]	Completed	Stereotactic aspiration with thrombolytics	March 1996–May 1999	13 centers in the Netherlands	36 Surgical vs. 35 Non-surgical	**Mortality:** 56% vs. 59% (*p* = 0.78) at 180 days **Functional Status:** no difference in likelihood of mRS >4 (OR = 0.52, *p* = 0.38) **Rebleeding:** 0% vs. 22%, *p* = 0.006
Minimally Invasive Surgery Plus Rt-PA for ICH Evacuation Phase III (MISTIE III) [39]	Completed	Stereotactic aspiration with thrombolytics	December 2013–August 2017	84 centers Australia, Canada, China, Germany, Hungary, Israel, Spain, UK, USA	255 MISTIE vs. 251 Standard medical care	**Mortality:** 19% vs. 26% (*p* = 0.04), at 365 days **Functional Status:** no difference in mRS <4 at 365 days (45% vs. 41%, = 0.33) **Rebleeding:** 2% vs. 1%, *p* = 0.32
Early Minimally-Invasive Removal of Intracerebral Hemorrhage (ENRICH)	Ongoing	Endoport	December 2016–December 2021	36 centers in USA	Expected enrollment: 300	n/a—study ongoing
Auer et al. 1989 [40]	Completed	Endoscope	June 1983–August 1986	Austria	50 Endoscopic evacuation vs. 50 Medical management	**Mortality:** 42% vs. 70% (*p* < 0.01) at 180 days **Functional Status:** significant difference in “minimal neurologic deficit” at 180 days (40% vs. 25%, *p* < 0.05) **Rebleeding:** 4% vs. 30%, *p* < 0.05
Intraoperative Stereotactic Computed Tomography-Guided Endoscopic Surgery (ICES) [41]	Completed	Endoscope	August 2005–August 2012	29 centers in Canada, Germany, USA, UK	14 Surgical vs. 4 Medical	**Mortality:** 0% vs. 7.1% (*p* = 0.68) **Functional Status:** no difference in mRS <4 at 180 days (42% vs. 24%, *p* = 0.19) **Rebleeding:** no rebleeding in either group
Minimally Invasive Endoscopic Surgery with Apollo in Patients with Brain Hemorrhage (INVEST)	Ongoing	Apollo	June 2017–June 2021	7 centers in USA	Estimated enrollment: 50	n/a—study ongoing
Artemis in the Removal of Intracerebral Hemorrhage (MIND)	Ongoing	Artemis	February 2018–July 2024	20 locations in Germany and USA	Estimated enrollment: 500	n/a—study ongoing
Dutch Intracerebral Hemorrhage Surgery Trial (DIST)	Ongoing	Artemis	November 2018–present	10 centers in the Netherlands	Estimated enrollment: 400	n/a—study ongoing
Minimally Invasive Intracerebral Hemorrhage Evacuation (MIRROR)	Ongoing	Surgiscope	October 2020–October 2028	2 centers in USA	Estimated enrollment: 500	n/a—study ongoing
Ultra-Early, Minimally Invasive Intracerebral Hemorrhage Evacuation Versus Standard Treatment (EVACUATE)	Ongoing	Surgiscope	September 2020–December 2025	2 centers in Australia	Estimated enrollment: 240	n/a—study ongoing

n/a—not applicable.

## Data Availability

Not applicable.
